# Implementation of Chemometrics and Other Techniques as Means of Authenticity and Traceability to Detect Adulteration in Foods for the Protection of Human Health

**DOI:** 10.3390/foods12030652

**Published:** 2023-02-02

**Authors:** Theodoros Varzakas

**Affiliations:** Department of Food Science and Technology, University of the Peloponnese, 24100 Kalamata, Greece; t.varzakas@uop.gr

The authenticity of foods of plant and animal origin is key to safeguarding both quality and safety aspects without jeopardizing consumers’ health. Hence, this Special Issue brings advances in the area of the authentication and chemometrics of foods, in attempt to prevent fraud, a phenomenon which has increased recently. Different food materials have been investigated and the results have been reported.

Tocopherols (α-, β-, γ-, and δ-) are part of the commonly known vitamin E complex, along with the corresponding tocotrienols, which protect against the non-enzymatic peroxidation of polyunsaturated fatty acids, are lipid-soluble, and can be found in high-fat plant foods, such as walnuts. In the first paper, Mitsikaris et al. [1] determined the levels of tocopherols in walnut seed oils by HPLC-UV. The levels of the samples varied from four European countries and showed that the Ukrainian walnut seed oils exhibited significantly higher total concentrations. Moreover, a higher mean concentration of α-tocopherol was reported for Greek walnuts compared to the French and Bulgarian walnuts.

Zhao et al. [2] discriminated the lamb meat quality of Protected Geographical Indication (PGI) Sunite lamb, from two other banners in the Inner Mongolia autonomous region by stable isotopes and modeling. They found that this methodology, in conjunction with chemometric approaches such as the data-driven soft independent modeling of class analogy (DD–SIMCA), can be used as an effective indicator for protecting PGI Sunite lamb, taking into account geographical origin, feeding system, age, and gender.

Rana et al. [3] discriminated the physico-functional properties and chemometric techniques (principal component analysis (PCA) and multiclass discriminant analysis (MDA)) of four *Cinnamomum* species. They found a clear separation of the different *Cinnamomum* species using the above techniques, thus showing that this combination is effective against food fraud.

Siddiqui et al. [4] employed Fourier infrared spectroscopy (FTIR) to determine adulteration in meat mixtures of beef, lamb, and chicken. They also used PCA and multiclass support vector machine (M-SVM) and found that the highest classification accuracy value of 85% was presented in beef and lamb samples for both adulterated and non-adulterated classes. This method could be a rapid quality control tool in the meat industry and might be employed in halal authentication. 

Shomaji et al. [5] reported the use of low-field ^1^H-nuclear magnetic resonance (NMR) relaxometry to determine dye contamination on vegetables. It is very well known that non-food-grade dyes could be very toxic. It was concluded that the proposed low-cost detection approach can be used to generate warning flags if the detected dye concentrations are over the limit of the accepted standards for food dyes.

The article by Agriopoulou et al. [6] discussed Greek table olive varieties from protected designation of origin (PDO) areas. The authors used orthogonal partial least square discriminant analysis (OPLS-DA) for the discrimination and classification of table olives and found the model to be effective in olive fruit authentication.

Zhao et al. [7] employed multivariate data analysis applied to elemental analysis, stable isotope analysis, and fatty acid analysis in combination with orthogonal partial least squares discriminant analysis (OPLS-DA) to determine the geographical origin of milk in four neighboring provinces. The discrimination of milk took place in farms with different distances of less than 11 km in each province, and the discriminant distance was successfully reduced to 0.7 km.

They found that for a relatively close sample origin distance, a single technique, such as the fatty acid chemical parameter analysis, could be completely superior compared to the combination multiple technologies. These results could be used to improve milk traceability in China.

The paper by Tarapoulouzi et al. [8] reported on stable isotope ratio analysis and orthogonal projections to geographically discriminate Greek olive cultivars. This combination was very good and they showed that the most important isotope markers for the discrimination of olive oil samples were δ^18^O and δ^2^H.

Tarapoulouzi et al. [9] discussed and reviewed the evolution of the use of chemometrics on honey composition/the physico-chemical parameters during processing and storage in order to determine the authenticity of honey. They verified it as an effective tool to optimizing quality control and the safety protection of consumers’ health.

Another review paper by Grassi et al. [10] compared the different chemometric techniques (from clustering to classification and regression) along with spectroscopy, chromatography, electrochemical sensors, and other on-site detection devices against milk adulteration. They also presented the steps which should be followed to develop a chemometric model to face adulteration issues.

Finally, Avila-Sosa et al. [11] determined the specific chemical markers coupled with chemometric methods to discriminate the adulterated samples of saffron. Saffron is an important colorant, antioxidant, and source of phytochemicals aromatic spice due to the large number of chemical compounds found in the by-products (flower parts) of saffron (catechin, quercetin, delphinidin, etc.). They found that the geographical origin and harvest/postharvest characteristics of saffron could play a key role in chemical characterization.

## Data Availability

Data is contained within the article.
